# The Risk of Relapse in Papillary Thyroid Cancer (PTC) in the Context of *BRAF^V600E^* Mutation Status and Other Prognostic Factors

**DOI:** 10.1371/journal.pone.0132821

**Published:** 2015-07-15

**Authors:** Agnieszka Czarniecka, Monika Kowal, Dagmara Rusinek, Jolanta Krajewska, Michal Jarzab, Ewa Stobiecka, Ewa Chmielik, Ewa Zembala-Nozynska, Stanislaw Poltorak, Aleksander Sacher, Adam Maciejewski, Jadwiga Zebracka-Gala, Dariusz Lange, Malgorzata Oczko-Wojciechowska, Daria Handkiewicz-Junak, Barbara Jarzab

**Affiliations:** 1 The Oncologic and Reconstructive Surgery Clinic, Maria Sklodowska-Curie Memorial Cancer Center and Institute of Oncology, Gliwice Branch, Gliwice, Poland; 2 Department of Nuclear Medicine and Endocrine Oncology, Maria Sklodowska-Curie Memorial Cancer Center and Institute of Oncology, Gliwice Branch, Gliwice, Poland; 3 III Dept. of Radiotherapy and Chemotherapy, Maria Sklodowska-Curie Memorial Cancer Center and Institute of Oncology, Gliwice Branch, Gliwice, Poland; 4 Department of Tumor Pathology, Maria Sklodowska-Curie Memorial Cancer Center and Institute of Oncology, Gliwice Branch, Gliwice, Poland; IPATIMUP/Faculty of Medicine of the University of Porto, PORTUGAL

## Abstract

**Introduction:**

The risk of over-treatment in low-advanced PTC stages has prompted clinicians to search for new reliable prognostic factors. The presence of *BRAF* mutation, the most frequent molecular event in PTC, seems to be a good candidate. However, there is still lack of randomised trials and its significance has been proved by retrospective analyses, involving a large group of patients. The question arises whether this factor is useful in smaller populations, characterised for specialised centres. Thus, the aim of the study was to evaluate the use of *BRAF* mutation as a potential predictive marker in PTC patients.

**Material:**

233 PTC subjects treated between 2004-2006, were retrospectively analysed. Stage pT1 was diagnosed in 64.8% patients and lymph node metastases in 30.9%. Median follow-up was 7.5 years. *BRAF^V600E^* mutation was assessed postoperatively in all cases.

**Results:**

*BRAF ^V600E^* mutation was found in 54.5%. It was more frequent in patients > 45 years (p=0.0001), and associated with larger tumour size (p=0.004). Patients with tumours <= 10 mm were over-represented among *BRAF* negative population (p=0.03). No association between *BRAF* mutation and other clinicopathological factors was observed. *BRAF* status was associated neither with relapse nor with disease-free survival (DFS) (p=0.76). Nodal status, extrathyroidal invasion and tumour size significantly influenced DFS.

**Conclusion:**

The risk of PTC recurrence is mainly related to the presence of lymph node metastases and extrathyroidal invasion, whereas no impact of *BRAF ^V600E^* mutation has been demonstrated.

## Introduction

The increasing incidence of thyroid cancer, especially low-risk stages has been recently observed worldwide [1,2,3,4]. The growing number of low-stage PTC has raised the discussion about the optimal therapeutic strategy, including the extent of surgery, indications to prophylactic central lymph node (LN) dissection and adjuvant radioiodine therapy [5–9]. The prognosis in differentiated thyroid cancer is generally good. However, about 10–15% of patients develop local or distant recurrences [8,10,11]. It is essential to create strategies of adequate patients stratification to avoid the risk of suboptimal treatment in high-risk patients [9,12–15] and simultaneously to prevent significant therapy de-escalation in patients with clinically indolent disease.

Searching for molecular markers is a possible way to achieve this goal. *BRAF* ^*V600E*^ mutation, being the most frequent oncogenic event and observed in about 50% of PTCs, is one of the best candidates [2,16,17]. This mutation, activating the MAPK pathway, plays a crucial role in malignant phenotype of PTC. The presence of *BRAF* mutation may be detected preoperatively, at the time of initial diagnosis from a fine-needle aspiration specimen, and thus it may influence the choice of further treatment strategy [5,12,15,18–21]. The prognostic importance of *BRAF* mutation has been analysed since the landmark studies, however, with controversial conclusions [7,10,12,22–26]. So far, there has been still lack of randomised trials supporting the prognostic significance of *BRAF* mutation. Recently published retrospective, multicentre analyses, involving a large group of PTC patients have demonstrated the association between *BRAF* mutation and both cancer-related mortality and PTC recurrence, albeit partially depending on other disease risk factors [27,28]. The question arises whether *BRAF* mutation is also useful as a prognostic factor in smaller populations, characterised for specialised surgical centres. Thus, the aim of this study was to evaluate the presence of *BRAF* mutation as a potential predictive marker in PTC patients and its possible association with disease prognosis with reference to other clinicopathological risk factors.

## Material and Methods

Two hundred thirty eight PTC patients diagnosed by fine needle aspiration biopsy were analysed in a retrospective manner (S1 Table). These patients were selected from the population of all patients treated surgically at the Department of Oncological and Reconstructive Surgery at Center of Oncology—M. Sklodowska-Curie Memorial Institute, Gliwice Branch, fulfilling the following criteria: 1) primarily operated between 2004–2006, 2) with FFPE material available for molecular analysis, 3) with PTC post-operative confirmation in histopathological assessment. The group consisted of 209 women (87.8%) and 29 men (12.2%). The presence of *BRAF* ^V600E^ mutation was evaluated in all subjects. Most PTC tumours (151; 63.4%) were diagnosed as T1. Lymph node involvement was observed in 77 (32.4%) subjects, whereas distant metastases in 8 (3.4%) patients. The mean time of follow-up was 7.1 years, median 7.5 years (range: 4 months to 9.93 years). Five patients, staged T4N1M1, diagnosed with disseminated PTC, were excluded from the further analysis. Three of them were *BRAF*(+) and 2 others *BRAF*(-). Among them 1 patient died due to thyroid cancer. It should be stressed that 88 patients in this group were previously described in a small pilot study in 2010 [29].

The detailed analysis involved 233 (25 males, 208 females) PTC subjects, M0 at diagnosis. Mean age was 46.2 year (median 46.1). All subjects underwent total thyroidectomy with routine central neck LN dissection. In 172 (73.8%) patients it was elective central lymphadenectomy in 61 patients (26.2%) therapeutic procedure was done. Additionally, unilateral LN neck dissection was performed in 43 patients (18.5%) and bilateral neck lymphadenectomy in 9 (4%) N1 cases. Two hundered and twenty seven (97.6%) patients received adjuvant radioiodine treatment. One hundred and ninety six (84.1%) patients received the single therapy. In 31 (11%) more than one was necessary. L-thyroxine with suppressive doses was administered to all patients with dose reduction when indicated.

During the further follow-up recurrent PTC was observed in 12 patients (5.15%): 9 patients developed local recurrence, whereas distant metastases were diagnosed in three T3 subjects (2 patients had both distant metastases and local relapse) (Table 1).

**Table 1:** Postoperative TNM staging in 233 PTC. M0 patients primarily operated on due to thyroid carcinoma between 2004 and 2006. Distant metastases were diagnosed during follow-up in three T3 patients.

T feature	All patients		N0 M0		N1 M0		M1	
	number	%	number	%	number	%	number	%
pT1	151	64.8%	119	78.8%	32	21.2%	0	--
pT1a	86	37%	75	87.2%	11	12.8%	0	--
Micro	66	28.3%	61	92.4%	5	7.6%	0	--
pT1b	65	27.8%	44	67.7%	21	32.3%	0	--
pT2	22	9.4%	16	72.7%	6	27.3%	0	--
pT3	53	22.8%	23	43.4%	27	50.9%	3	5.7%
pT4	7	3%	3	42.9%	4	57.1%	0	--
All	233	100%	161	69.1%	69	29.6%	3	1.3%

### Ethics Statement

The study was conducted after the approval of the Bioethics Committee MSC Memorial Cancer Center and Institute of Oncology, Gliwice Branch. Informed written consent was obtained from all patients or caregiver for the use of their tissue for analysis in this study. All clinical data were anonymized and de-identified prior to analysis.

The evaluation of *BRAF* mutation was done after DNA extraction from 10 μm-thick sections of FFPE tissues (5 sections per block) preceded by deparaffinisation using a single xylene extraction and rinsing with 98% ethanol. DNA isolation was performed with the Qiagen DNeasy Blood & Tissue Kit, according to the manufacturer’s protocol (Qiagen GmbH; Hilden, Germany). DNA concentration was evaluated with the use of the Nanodrop ND-100 microspectrophotometer. PCR was performed with primers spanning *BRAF* codon 600: F-5’-tgttttcctttacttactacacctca-3’ and R- 5’-gcctcaattcttaccatcca3’. The PCR product was than analysed with the Sanger’s direct sequencing method on the 3130xl Genetic Analyzer Applied Biosystems (Life Technologies, Carlsbad CA, USA).

Statistical analysis was performed with the use of IBM SPSS Statistics 22 and JMP 10 (SAS Institute, Cary, NC, USA) software. The analysis of survival data was performed using Kaplan-Meier method, with log-rank test comparison between subgroups. Multivariate survival analysis was done by the Cox method. Continuous variables were analysed with non-parametric U Mann-Whitney test, whereas associations of categorical variables were assessed by the exact Fisher test. Classification and Regression Trees were used to visualise the data.

## Results

### The frequency BRAF mutation

**Fig 1:**
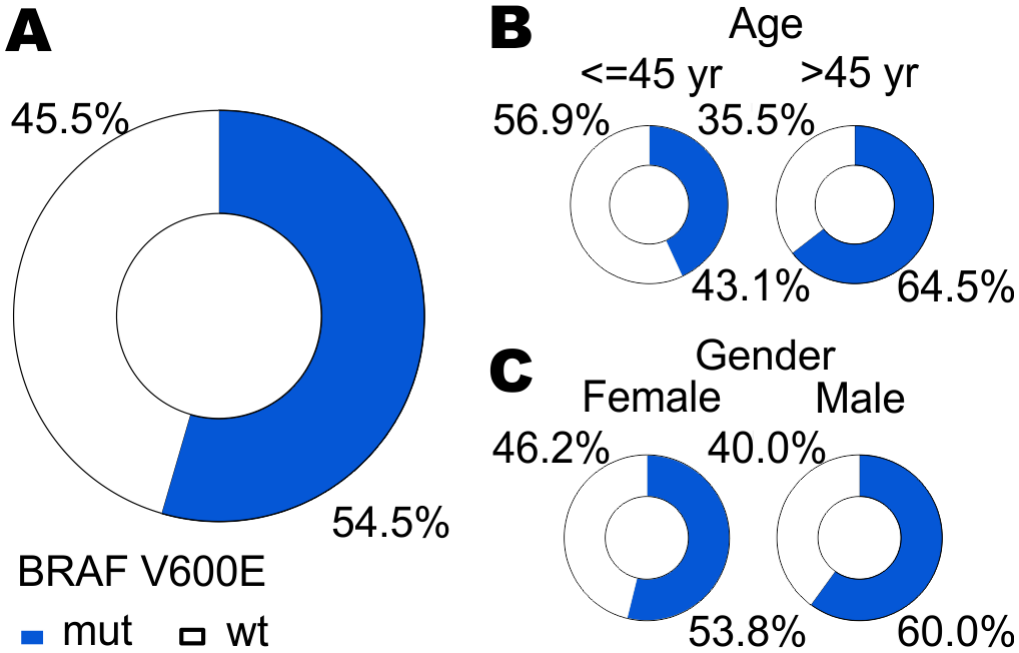
The occurrence of *BRAF* mutation in the whole study population (A), according to patients’ age (B) and gender (C). MUT—BRAF-positive samples; WT—BRAF-negative samples.

*BRAF* ^V600E^ mutation was found in 127/233 (54.5%) PTC patients (Fig 1). *BRAF* mutation was more frequent in older patients (64.5%) as compared to the younger subgroup, under the age of 45 years (56.9%) (p = 0.0001). The mean age in BRAF (+) group was 49.6 years (median: 51.1 years) whereas in BRAF (-) 42.0 years (median: 41.4 years), respectively. Patients with *BRAF* mutation were older than patients without the mutation (p<0.01). It was present slightly more frequently in men (60%) than in women (53.4%), although the difference was insignificant, probably due to the low number of male subjects (p = 0.67) (Fig 1).

### The association between BRAF mutation and tumour size

**Fig 2:**
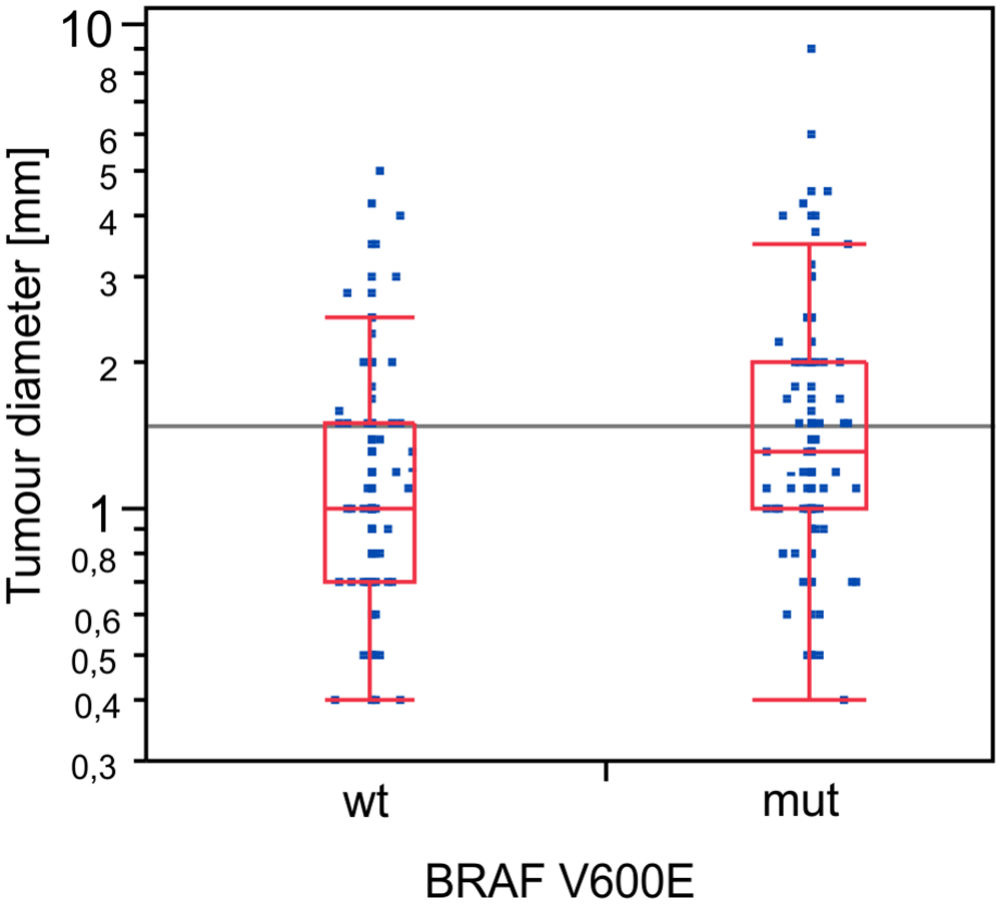
The average tumour size in *BRAF* positive patients was significantly higher than in the wild-type group (p = 0.04).

*BRAF* ^*V600E*^ mutation was associated with larger tumour size (Fig 2). The average tumour size in *BRAF*(+) patients was only slightly larger than in *BRAF*(-) group (16.5 mm vs 13 mm, respectively; the median values 13 mm vs 10 mm, p = 0.004). When pT1 stage was considered (< = 20 mm), there was no significant difference between *BRAF*(+) and (-) group, 62.2% and 75.8%, respectively (p = 0.11). Conversely, subjects both with tumours < = 10 mm in diameter and microcarcinoma (unifocal pT1a) were over-represented among *BRAF*(-) population: in *BRAF*(-) tumours there were 47 patients with tumour size to 10 mm (49.5%) vs 39 among *BRAF*(+) (30.7%, p = 0.03). Papillary microcarcinomas constituted 40% of the *BRAF*(-) group (38 patients) and 22% of the *BRAF* (+) group (28 patients, p = 0.01) (Table 2).

**Table 2:** The association between *BRAF* mutation and TNM classification and other pathological factors.

Factor	*BRAF*(+)		*BRAF*(–)		p
Whole group	n = 127	(54%)	n = 106	(46%)	
T1	79	62.2%	72	75.8%	p = 0.36
T1a	39	30.7%	47	49.5%	p = 0.03
microPTC	28	22%	38	40%	p = 0.01
T1b	40	31.5%	25	26.3%	p = 0.18
T2	14	11%	8	7.5%	p = 0.36
T3	30	23.6%	23	21.7%	p = 0.72
T4	4	3.1%	3	2.8%	p = 0.88
N1	34	26.8%	38	35.8%	p = 0.13
M1	1	0.8%	2	1.9%	p = 0.45
Multifocality	40	31.5%	37	34.9%	p = 0.58
Thyroid capsule invasion	34	26.8%	26	24.5%	p = 0.69
Vascular invasion	5	4%	6	5.7%	p = 0.54

### The association between BRAF mutation and other clinicopathological factors

We observed no association between the *BRAF* status and multifocality (31.5% of *BRAF*(+) vs 34.9% of *BRAF*(-)), thyroid capsule invasion (26.8% in *BRAF*(+) and 24.5% in *BRAF*(-), vascular invasion (4% in *BRAF*(+) and 5.7% in *BRAF*(-)) LN metastases (26.8% in *BRAF*(+) and 35.8% in *BRAF*(-) or distant metastases (0.8% in *BRAF*(+) and 1.9% in *BRAF*(-)) (Table 2).

### The association of cancer relapse with BRAF status and other clinicopathological factors

Cancer relapse was diagnosed in 12/233 (5.15%) patients. After 5-year follow-up 93% patients were still disease-free. No deaths occurred in the analysed group.

**Fig 3:**
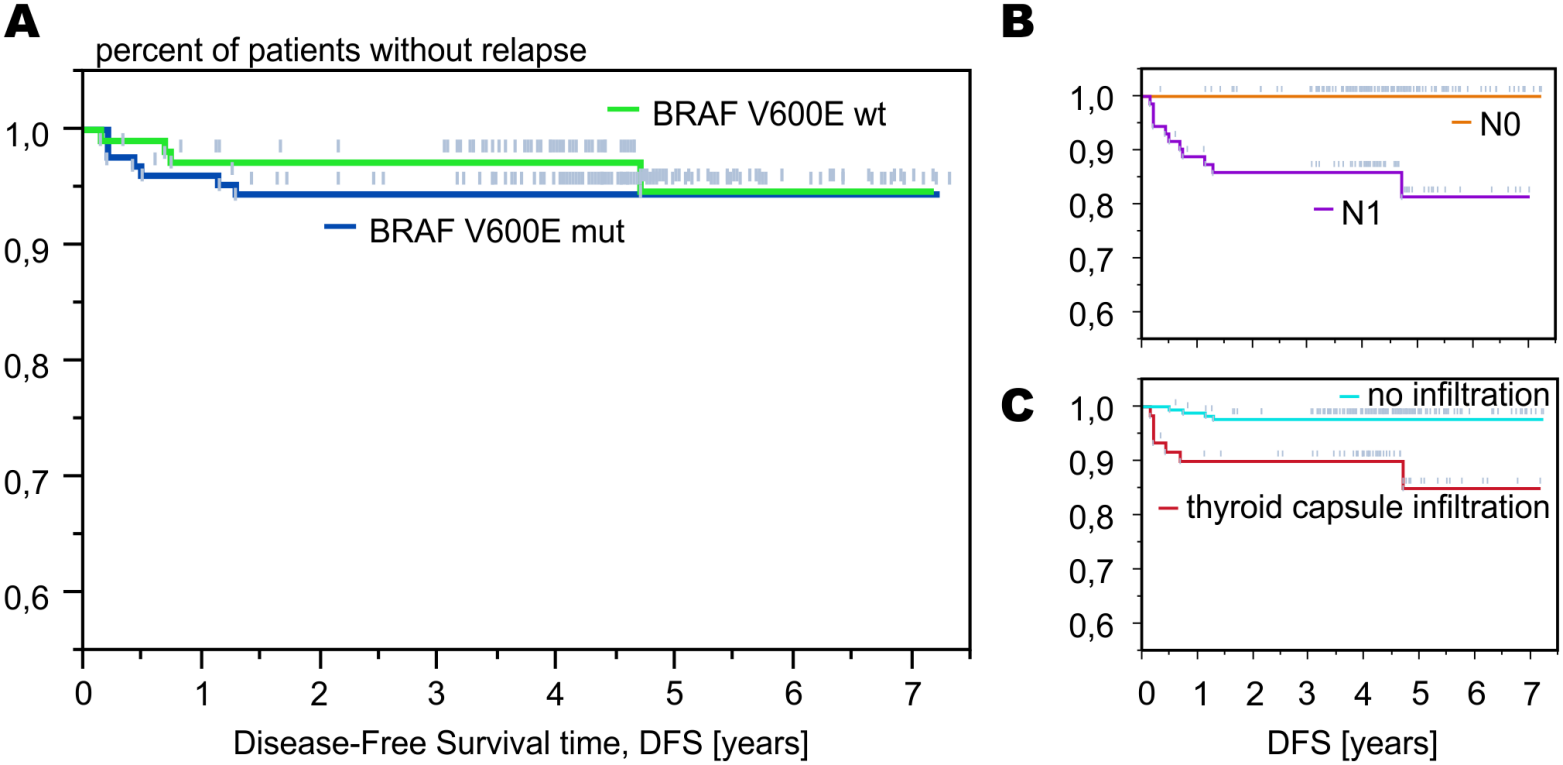
The presence of *BRAF* mutation did not increase the risk of cancer relapse (A). However, known histopathological factors, such as lymph node involvement (B) and thyroid capsule infiltration (C) significantly influenced disease-free survival.

*BRAF* status was associated neither with the cancer relapse nor with the time to relapse. Within the *BRAF*(+) group 7 cancer recurrences were noted (5.5%), including 6 local relapses and 1 distant metastases, whereas in the *BRAF*(-) group 5 cancer recurrences (4.7%) were reported, including 3 local relapses and 2 cases of local relapse with concurrent metastatic disease. There was no difference in DFS between *BRAF*(+) and *BRAF*(-) groups (Fig 3; p = 0.76).

However, the nodal status was highly associated with cancer relapse (Fig 3). There were no relapses in N0 patients, whereas all 12 (16.7%) were diagnosed in N1 group. All recurrences occurred in patients with macrometastases to the lymph nodes. In all of them unilateral or bilateral metastases to the cervical lymph nodes were noted. In 7 patients (58%) extranodal extension (extracapsular extension of lymph node metastases) was observed. All of them received radioiodine therapy. In almost all cases complete remission of the disease was reached. (Table 3).

**Table 3:** Detailed description of all cancer relapses.

BRAF positive								
Gender (Age–years)	pT	pN	pM	Central neck dissection	Lateral neck dissection	Extranodaldisease	Number of ^131^I therapy (mCi dose)	Outcome
F (26)	3	1b	0	therapeutic	Bilateral	Yes	4 (400)	CR
F (26)	3	1b	0	therapeutic	Unilateral	Yes	2 (200)	CR
F (56)	3	1b	1 *	therapeutic	Unilateral	Yes	2 (200)	SD
M (59)	4	1b	0	therapeutic	Unilateral	Yes	2 (200)	CR
F (29)	1b	1b	0	therapeutic	Unilateral	No	1 (100)	CR
F (51)	1b	1b	0	prophylactic	Unilateral	Yes	2 (200)	CR
F (49)	1a	1b	0	prophylactic	Unilateral	No	2 (200)	CR
BRAF negative								
F (42)	3	1b	0	therapeutic	Bilateral	Yes	2 (200)	CR
M (26)	3	1b	0	therapeutic	Unilateral	No	1 (100)	CR
F (46)	3	1b	1**	therapeutic	Unilateral	No	4 (400)	CR
F (21)	3	1b	1**	therapeutic	Unilateral	Yes	4 (400)	CR
F (35)	1b	1b	0	prophylactic	Unilateral	No	2 (200)	CR

F-female, M-male, CR- complete remission, SD- stable disease

* lung metastases without radioiodine uptake

** ^131^I positive lung micro-dissemination

A strong association was also observed with reference to extrathyroidal invasion: 5-year DFS in patients without and with thyroid capsule infiltration was 98% and 83%, respectively (p = 0.002; Fig 3). Tumour diameter significantly correlated with the risk of relapse—larger tumour size increased the risk, with hazard ratio of 1.39 per 10 mm of diameter (p = 0.033). There was a trend towards the association of age with prognosis: per 10 years of age we observed 0.7 reduction of risk of relapse (HR 0.696, 95% CI 0.48–1.01). No difference was observed between patients younger and older than 45 years when these two groups were compared. Additionally, multifocal tumour growth (p = 0.99), vascular invasion (p = 0.23) and gender (p = 0.47) did not influence the outcome.

### The association of cancer relapse with BRAF status and radioiodine therapy

Additionally, we did not observe the differences in the cumulative radioiodine activity given (p = 0.55) or the number of ^131^I therapy courses (p = 0.73) to reach disease-free status between BRAF(+) and BRAF(-) groups. In the BRAF(+) group 110 patients (86.6%) were in complete remission after a single ^131^I-therapy (dose of 100 mCi) and 86 (81.1%) patients in BRAF(-). 7 cancer relapses were observed in the BRAF(+) group 1 patient received single therapy, 4 patients two therapy courses and 1 patient 4 radioiodine treatments. Complete remission was achieved in 6 patients after treatment. One patient with pulmonary metastases without radioiodine uptake received the dose of 200 mCi and presented with stable disease. (Table 3)

In the BRAF(-) group 5 cancer recurrences were noticed. One patient received single therapy, 2 patients double and 2 patients 4 treatments. Complete remission was achieved in all patients in the BRAF(-) group (Table 3).

### Association of LN metastases with other clinical parameters

Among the other risk factors related to LN metastases, confirmed in the univariate analysis were extrathyroidal invasion (p = 0.00), multifocal tumour growth (p = 0.02) and larger tumour diameter (p = 0.00). Lymph node metastases occurred significantly more frequently in patients with larger tumour diameter (HR 1.68 per 10 mm of tumour diameter), whereas their probability decreased with patient age (HR 0.68 per 10 years of age). N1 patients were younger (mean age 41 years, median 36 years) than subjects without nodal involvement (mean age 49 years, median 52 years, p<0.001). Tumour size was larger in N1 patients (mean diameter 19 mm, median diameter 15 mm) than in N0 subjects (mean diameter 13 mm, median diameter 10 mm, p<0.001). However, there was no relationship between LN metastases and *BRAF* status (p = 0.13), gender (p = 0.29) and vascular invasion (p = 0.18).

### Multivariate analysis

**Fig 4:**
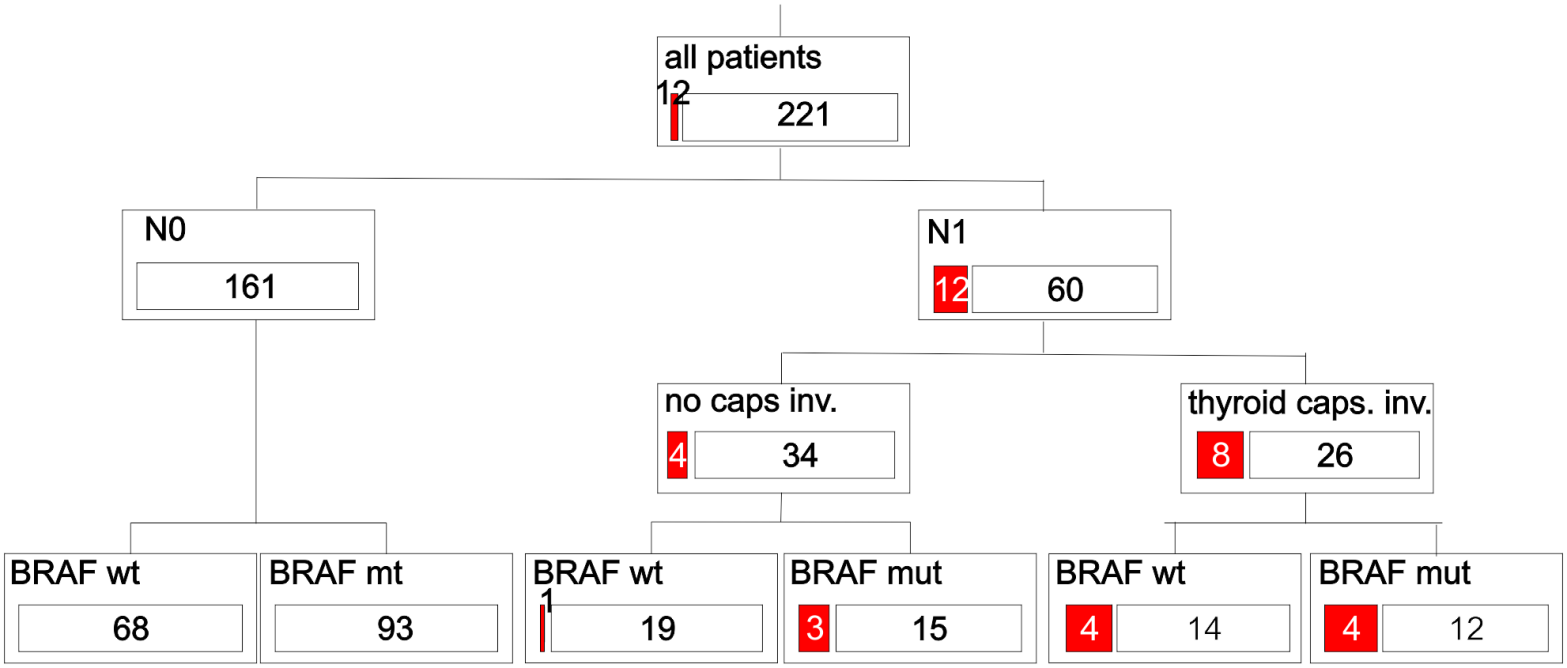
Classification and regression tree analysis (CART) for discrimination between patients with relapse (denoted by a red bar) and non-relapsing patients (white bars). Numbers of patients are given, proportion of bars is proportional to the number of patients.

We attempted to perform the multivariate analysis. However, as described above, in the subgroup of patients with no nodal metastases (N0) there was no single event of cancer relapse, thus the analysis of the association with clinical parameters and *BRAF* status in multivariate setting was impossible if nodal metastases have been considered a variable in the model. Simultaneously, both in our group and other studies the prognostic importance of nodal status is so strong that this factor could not be omitted. Thus, we attempted to analyse and visualise the data by the CART method. Although the subsets are too small to provide significant associations, it could be noticed that both nodal status and thyroid capsule infiltration independently provide some significant information on relapse. The relapse risk is slightly higher in *BRAF*-mutated patients in comparison to *BRAF* wild type patients, although the minor differences are not significant in these small subsets (Fig 4).

When multivariate Cox regression was performed in the subgroup of N1 patients, only thyroid capsule invasion was influencing the risk of relapse (HR 5.1, p = 0.014). No association of *BRAF* status was found in multivariate analysis in this N1 subgroup.

## Discussion

Currently, there are contradicting views on the role of *BRAF* mutation as a risk predictor in PTC. In our study the prognostic impact of *BRAF* mutation in a group of mainly early-stage PTC patients is weak and cannot be detected. Its prognostic significance has been a matter of debate for 10 years [7,10,12,14,22,23,25,26,30,31]. Due to relatively low mortality rate in PTC, especially in low advanced cases with excellent therapy outcomes, the majority of studies concentrate on the association between *BRAF* status and disease-free survival [7,23,25,32,33]. Kebebew at *al*. [7] proved that *BRAF* mutation was independently associated with recurrent and persistent disease. In the multivariate analysis, the presence of *BRAF* mutation and LN metastases independently influenced unfavourable outcome. In contrast to their study, we did not observe the impact of *BRAF* status on DFS, yet our study demonstrated a significant correlation between poor outcomes and LN status or extrathyroidal invasion.

Although the association of *BRAF* with poor prognosis was not observed in the present study, numerous reports show the influence of *BRAF* mutation on patients’ prognosis even in the low-risk PTC [12,30,31,34]. This finding is especially important as most PTCs are currently diagnosed in a low stage. Also in our study almost 75% of patients were T1–T2 at the time of diagnosis.

However, numerous papers fail to demonstrate a correlation between *BRAF* mutation and prognosis [21,35,36], including the studies carried out in Korean and Japanese populations where the incidence of *BRAF* mutation is higher than in Europe and the US (70–80%) [10,18,35,37–39].

The differences among the published data might be related to cohort size, time of follow-up, relapse rate and distinct treatment algorithms in particular countries. For instance, in a study involving 110 PTC patients, follow-up was relatively short (8 months) and no impact of *BRAF* mutation on cancer stage and prognosis was demonstrated [40]. In our group only 5 relapses (42%) occurred during the first 8 months of observation, thus such duration may limit the possibility of drawing conclusions. In contrast, no relapses occurred in our group later than 5 years, which is consistent with typically good outcome of early stage PTC.

Depending on the country, national recommendations vary with reference to the extent of surgery, especially indications to prophylactic central neck dissection and adjuvant radioiodine treatment [41–44]. Our population, underwent total thyroidectomy with routine central neck dissection, followed by adjuvant radioiodine therapy and thyroid hormone suppression. After 7-year follow-up the overall relapse rate was only 5.2% (12/233) in the whole cohort and 5.5% (7/127) in BRAF(+) patients. The intensive initial treatment might be a potential reason explaining why no association of *BRAF* mutation was found as patients with worse prognosis were subjected to all treatment modality. Furthermore, a considerable majority of low-advanced tumours did not allow to demonstrate lower sensitivity to radioiodine therapy in BRAF(+) patients.

In our study the main risk factor influencing DFS was LN status. Recently more attention has been paid to the significance of the advancement of nodular disease for the prognosis in PTC patients [45]. It is well known that most cases of local recurrence are non-radioiodine-avid LN metastases [5,7]. Therefore, some authors try to find any correlation between *BRAF* mutation and the risk of LN involvement [5,9,11,12,46,47]. Unfortunately, we did not observe a significant relationship between *BRAF* mutation and LN status. Only a slight and insignificant trend towards an increased relapse rate was noticed when the presence of *BRAF* mutation was considered with reference to LN metastases and extrathyroidal invasion. However, Joo et *al*. [13] observed (both in univariate and multivariate analyses) an association between *BRAF* mutation and a higher risk of central neck LN metastases. The authors conclude that *BRAF* mutation diagnosed by FNAB should be considered a factor influencing the extent of surgery. Others authors also reported a similar relationship between *BRAF* mutation and the risk of LN metastases and suggested using this mutation as a prognostic factor [9,11,18,36]. Contrary to these reports, there are some papers that as in our analysis, did not confirm any association of *BRAF* mutation and other risk factors, including LN metastases [15,21,25,38,46]. This discrepancy requires a prospective study, however, the feasibility of such a trial although questionable has been shown [48]. Paradoxically, generally good prognosis in PTC and its long-standing clinical course significantly impedes searching for an unequivocal response related to optimisation of a therapeutic strategy. In 2012, an attempt to estimate the proper number of patients necessary to perform a multicentre, prospective, randomised study with reference to prophylactic central neck dissection was performed by the ATA. Such a study would require about 6 000 patients and at least 7-year follow-up, which is considered an unreachable aim [49].

Meta-analyses, involving large PTC populations, constitute an indirect way to resolve this problem [5,27]. In 2007, Lee et *al*. analysed 12 studies and concluded that *BRAF* mutation was a useful prognostic biomarker associated with advanced clinical stage, extrathyroidal invasion and histologic subtype [50]. Next, in 2012 Li et *al*. reviewed 32 studies and found that *BRAF* mutation correlated with poor prognostic factors, such as LN metastases, advanced stage, extrathyroidal invasion, tumour size, male gender and histological subtypes. However, the authors did not analyse the influence of *BRAF* mutation on prognosis in PTC [51]. In 2013, Xing et *al*. in a large retrospective multicentre study proved that the presence of *BRAF* mutation was significantly associated with cancer-related mortality, but this association was not independent of others risk factors, because of a low overall mortality in PTC patients [27]. Additionally, Xing et al. in 2015 demonstrated its association with poorer recurrence-free probability and increased risk of PTC relapse. The authors also noticed the highest risk of recurrence with the co-existence of the mutation and neck LN metastases [28]. It should be stressed that the pilot population of this study (88 patients) was included in these analyses [29].

The most important argument against an independent prognostic role of *BRAF* mutation [10,25,36] relates its overall incidence in PTC (about 30–80%) to the frequency of poor-prognosis patients (no more than 20%). In fact, *BRAF* mutation is probably not a single driver of aggressive PTC phenotype and may influence disease stage and treatment. Considering the complexity of an interaction between disease stage and therapy, the retrospective analysis of patient cohorts may be severely biased if these cohorts were not managed in the unified pattern. Moreover, the future retrospective studies should be targeted into uniform populations of patients, both in the context of disease extent and the treatment applied.

In fact, the results of meta-analyses confirming the role of *BRAF* mutation as an independent prognostic factor [27,28] are not in contrast with the results obtained in our study. The relatively low number of patients with cancer relapse in our cohort precludes any definitive conclusions, although we observe a slight and insignificant trend towards the increased relapse rate when *BRAF* mutation is considered with reference to LN metastases and extrathyroidal invasion. However, we decided to report these data as the bias towards positive-report publishing [52,53] may influence also the results of meta-analyses.

Considering the present knowledge and a question posed by Puxeddu [2] whether we are ready to implement the routine assessment of *BRAF* mutation into a clinical practice, we stress the importance of further research on the role of *BRAF* ^*V600E*^ mutation in the context of other prognostic factors [5,25,30]. Considering the equivocal opinions [5,12,18,20,31], at the moment this marker by itself will not dictate clinical decisions.

Studies, linking the importance of *BRAF* mutation and other molecular prognostic factors, such as *TERT* mutations or miRNA profile have been recently published [54–57]. A very interesting and promising observation was performed by Xing et al. [58]. These authors proved that co-existing *BRAF* ^V600E^ and *TERT* ^C228T^ mutations were more commonly associated with high-risk clinicopathologic characteristics of PTC and such co-existence may better define PTC with the unfavourable outcomes, providing unique prognostic and therapeutic implications. Thus, understanding of the complexity of PTC will probably increase in time and novel parameters may modify the diagnostic approach to PTC.

## Conclusions

To conclude, the risk of PTC recurrence is related to the presence of LN metastases and extrathyroidal invasion. The relationship between *BRAF* mutation and cancer relapse in mainly low-stage PTC population is not significant. A high prevalence of *BRAF* mutation, contrasted to very low relapse rate in the analysed group suggests that in early PTC *BRAF* mutation may be only one of the potential molecular prognostic factors and cannot be used as a sole determinant of prognosis.

## Supporting Information

S1 Table Patients information. (TXT)
